# Polymorphism rs5498 of the ICAM-1 gene affects the progression of carotid atherosclerosis in patients with type 2 diabetes mellitus

**DOI:** 10.1186/s12944-016-0247-y

**Published:** 2016-04-18

**Authors:** Dražen Popović, Jovana Nikolajević Starčević, Marija Šantl Letonja, Jana Makuc, Andreja Cokan Vujkovac, Ruda Zorc Pleskovič, Ludovit Gaspar, Peter Kruzliak, Danijel Petrovič

**Affiliations:** General Hospital Rakičan, Murska Sobota, Slovenia; Institute of Histology and Embryology, Faculty of Medicine, University of Ljubljana, Ljubljana, Slovenia; General Hospital Slovenj Gradec, Slovenj Gradec, Slovenia; 2nd Department of Internal Medicine, Faculty of Medicine, Comenius University and University Hospital, Bratislava, Slovakia; Laboratory of Structural Biology and Proteomics, Faculty of Pharmacy, University of Veterinary and Pharmaceutical Sciences, Palackeho tr. 1946/1, 612 42 Brno, Czech Republic

**Keywords:** ICAM 1, Polymorphisem rs5498, Carotid atherosclerosis, Type 2 diabetes mellitus

## Abstract

**Background:**

Adhesion molecules are involved in the development of atherosclerosis. An increased level of the ICAM 1 molecule is associated with numerous inflammatory diseases including atherosclerosis of carotid arteries. The rs5498 (K469E) polymorphism of the ICAM-1 gene leads to an increase in the level of serum ICAM. We investigated the association between the rs5498 (K469E) polymorphism of the ICAM-1 gene and the progression of carotid atherosclerosis in subjects with type 2 diabetes mellitus (T2DM).

**Methods:**

The study included 595 patients with T2DM and 200 subjects in the control group without T2DM. The control examination was made 3.8 years after the initial examination. Indicators of atherosclerosis (carotid intima-media thickness (CIMT), total plaque sum and sum of the plaques thickness) were detected by ultrasound examination. Genetic analyses of the polymorphism rs5498 of the ICAM-1 gene were made by RT-PCR.

**Results:**

The distribution of genotypes and frequencies of rs5498 polymorphism was not significantly different between the group with type 2 diabetes ( T2DM) and the control group. Genotype EE K469E polymorphism is associated with a statistically significant annual plaques growth.

**Conclusion:**

The EE genotype of the rs5498 of the ICAM-1 gene was associated with a more rapid progression of carotid atherosclerosis in patients with T2DM in comparison with other genotypes.

## Background

Atherosclerosis is a chronic inflammatory disease [1]. Type 2 diabetes mellitus (T2DM) is a chronic metabolic disease. In patients with diabetes, cardiovascular complications are reported about 15 years earlier than in the population without T2DM [2]. It has not been clarified how T2DM affects the development of atherosclerosis. It was reported that an increase of inflammatory cytokine levels (IL-6, IL-18 and TNF- ɑ) might cause plaque destabilization [3].

The leukocytes adhesion and their transendothelial migration play an important role in the initial phase of atherogenesis [4]. Processes are regulated by the various types of adhesion molecules, such as the intercellular adhesion molecule 1 (ICAM-1), the vascular cell adhesion molecule 1 (VACAM 1) and the platelet endothelial cell adhesion molecule 1 (PECAM 1). Their expression takes place on the surface of endothelial cells, hematopoetic cells, and immunological cells [5]. The plasma level of adhesion molecules is elevated in individuals with atherosclerosis [6–8]. The level of adhesion molecules in the serum is also elevated in patients with T2DM [9].

ICAM 1 is a transmembrane glycoprotein receptor consisting of 505 amino acids, with molecular weight between 80 and 114 KDa depending on the degree of glycosylation [10]. ICAM 1 belongs to the immunoglobulin superfamily. It acts as a ligand β 2-integrin present on leukocytes. It consists of five extracellular domains, a transmembrane domain and a short cytoplasmic domain [11].

Increased concentrations of sl ICAM-1 were repored in patients with cardiovascular disease, tumors, autoimmune disease and other diseases with an inflammatory reaction [7, 12, 13]. The gene for ICAM-1 is located on the short arm chromosome 19. It consists of 15 775 base pairs, and it has seven exons and six introns comprising a relatively large non-translation region [14]. Translational start region is located in exon 1 [14].

The literature describes two polymorphisms of the gene for ICAM 1, which affect the expression, K469E (rs 5498) and G241A (rs1799969) [15].

The rs5498 polymorphism in exon 6 of the gene for ICAM-1 affects the structure of ICAM 1 and, consequently, the migration of leukocytes in inflammatory milieu [16]. Motawi et al. found an increased incidence of E alleles K469E polymorphism of ICAM 1 in patients with peripheral and cardiovascular disease compared with the control group [17].

Bjelinski et al. have found an association between the K469E polymorphism and sl ICAM 1, however, there was no correlation between calcium in coronary arteries and circulating levels of sl ICAM 1 [18]. Milutinović and Petrovič reported that the EE and KK genotype polymorphism K469E for ICAM 1 was not associated with myocardial infarction in patients with T2DM [19]. An increased level of sl ICAM 1 in the healthy population represents a risk for future heart attacks according to Ridker et al. [13]. Those studies do not give a clear answer on the relationship between the K469E polymorphism of ICAM 1 and the development of cardiovascular diseases.

The purpose of the study was to investigate an association between the rs5498 polymorphism of the ICAM-1 gene and the progression of carotid atherosclerosis in patients with T2DM.

## Methods

### Subjects

The study included 595 patients with T2DM and 200 subjects without T2DM. Patients with T2DM were selected from diabetic outpatient departments of the General Hospital Murska Sobota and General Hospital Slovenj Gradec.

Control ultrasound examinations of the neck artery were performed on 426 patients with T2DM and on 132 subjects in the control group. The period between the first and the control ultrasound examination was 3.8 ± 0.5 years.

Patients with myocardial infarction (MI) and ischemic stroke were not enrolled in the study. The study was approved by the National Medical Ethics Committee.

### Ultrasound examination

High resolution B mode ultrasound analysis was performed using the portable ultrasound system Toshiba Aplio SSA-700 (Toshiba Medical. System Corp., Tokyo, Japan) connected to a multi-frequency linear array transducer. All examinations were performed by a single expert radiologist. Patients were examined in the supine position with the head tilted backwards. CIMT, defined as the distance from the leading edge of the lumen-intima interface to the leading edge of the media-adventitia interface, was measured at three sites along the 10 mm long segment of the far wall of the common carotid artery (CCA) free of plaques in agreement with the carotid intima-media thickness consensus [20]. CIMT on the left and on the right were calculated as the mean of three readings, and the mean of the left and right CCA-CIMT measurements was used in the analysis. For the purpose of logistic regression analysis we divided all the patients into two subgroups according to the median CIMT value.

### Biochemical analysis

Blood samples for biochemical analyses: fasting glucose, glycated haemoglobin (HbA1c), total cholesterol, triglyceride levels, high-density lipoprotein (HDL), low-density lipoprotein (LDL) cholesterol level were collected after a 12 h fasting period. All blood biochemical analyses were determined by standard biochemical methods in the hospital’s accredited lab.

### Genotyping

Genomic DNA was extracted from 100 μL of whole blood using a FlexiGene DNA isolation kit, in accordance with the recommended protocol (Qiagene GmbH, Hilden, Germany). The rs5498 polymorphism of the ICAM-1 gene was determined with real-time PCR using StepOne™ (48-well) Real-Time PCR Systems (Applied Biosystems, Foster City, CA, USA). We used the commmercially available genotyping kit TagMan SNP Genotyping assay (Applied Biosystem, Foster City, CA, USA) following the manufacturer’s instructions. Moreover, we used the following primers:-5’-CCATCGGGGAATCAGTG-3’;- 5’-ACAGAGCACATTCACGGTC-3’).

### Statistical analysis

Normality of distribution continuous variables was examined by using the Kolmogorov-Smirnov test. Continuous variables which were normally distributed, showed in the form of means ± standard deviations. Variables that were not in normal distribution, were presented in the form of median (interquartile range). To compare the numerical values of the continuous variables, we used Student’s *t* test or analysis of variance (ANOVA), if the variables were normally distributed. Mann–Whitney *U* test or Kruskal-Wallis H test if the variables were asymmetrically distributed. To compare frequencies categorical variables, statistical evaluation of differences in the frequencies of different alleles and genotypes between the two groups as well as in the case of determining the Hardy-Weinberg equilibrium, were used χ2 test.

The correlation between independent variables were analyzed using the Pearson correlation analyzes. The results showed a high degree of correlation between the serum levels of total and LDL cholesterol (*r* = 0.86; p <0.001) as well as systolic and diastolic blood pressure (*r* = 0.65, *p* <0.001). In the cases of a high degree correlation between the two variables only one variable from each pair were included in the multivariate statistical models. Change in the value of ultrasound markers of carotid artery atherosclerosis was calculated by deducting between value measured at two ultrasound examination.

For searching association between polymorphisms of selected candidate genes and their interactions with statins treatment and indicators of progression carotid artery atherosclerosis were used multivariate linear regression analysis. The criteria for a statistically significant difference is p value less then 0.05. To reduce the possibility of error due to the small number of subjects were used Bonferronis correction. All statistical analyzes were performed using a computer program SPSS for Windows, version 20 (Statistical Package for the Social Sciences Inc., Chicago, IL, USA).

## Results

Basic clinical characteristics and biochemical laboratory results are shown in Table 1. There were no statistically significant differences in age, body mass index, systolic and diastolic blood pressure between the group with T2DM and the control group. Waist circumference was significantly higher in the T2DM group, as well as the number of smokers (Table 1). A biochemical examination in patients with T2DM showed statistically significant higher levels of fasting glucose, HbA1c, total cholesterol, HDL, LDL, triglyceride and CRP-a compared with the control group (Table 1).

**Table 1:** Initially clinical and biochemical characteristics patients with T2DM and control subjects

	Patients with T2DM *n* = 595	Subjects without T2DM *n* = 200	*p*
Age	61.38 ± 9.65	60.07 ± 9.18	0.07
Male sex (%)	338 (56.8)	92 (46.0)	0.008
Duration T2DM	11.25 ± 7.88	-	**-**
Smoking (%)	53 (8.91)	34 (17.0)	0.002
Waist circumference (cm)	108.65 ± 12.88	93.31 ± 13.18	<0.001
BMI (kg/m2)	30.96 ± 4.74	27.90 ± 4.42	0.16
systolic pressure (mm Hg)	146.98 ± 19.98	143.3 ± 16.6	0.86
Diastolic pressure (mm Hg)	85.75 ± 11.62	84.7 ± 11.6	0.19
Fasting glucose (mmol/L)	8.04 ± 2.57	5.27 ± 0.87	<0.001
HbA1c (%)	7.89 ± 3.56	4.79 ± 0.29	<0.001
Total cholesterol (mmol/L)	4.70 ± 1.19	5.36 ± 1.08	<0.001
HDL cholesterol (mmol/L)	1.19 ± 0.35	1.43 ± 0.37	<0.001
LDL cholesterol (mmol/L)	2.63 ± 0,94	3.24 ± 0.98	<0.001
Triglycerides (mmol/L)	1.9 (1.2–2.7)	1.3 (0.9–1.9)	<0.001
High sensitivity CRP (mg/L)	2.2 (1.0–4.3)	1.3 (0.8–2.7)	<0.001

A control ultrasound examination of the carotid artery was made 3.8 ± 0.5 years after the initial examination. The progression of atherosclerotic markers (change in annual CIMT, change in the number of plaque segments, and change in the sum of the plaque thickness) was more intense in subjects with T2DM in comparison with subjects without T2DM (Table 2).

**Table 2:** Changes in echo markers of carotid atherosclerosis in patients with T2DM and control subjects between the first and control echo examination after 3.8 ± 0.5 years

	Patients with T2DM *n* = 426	Subjects without T2DM *n* = 137	*p*
Annual CIMT increment (μm/year)	20.33 (11.74–29.86)	12.83 (8.82–20.66)	0.02
Δ number of plaque segments	2.0 (1.0–3.0)	1.5 (0.7–2.2)	0.03
Δ sum of the plaques thickness (mm)	5.40 (2.40–7.05)	3.64 (2.88–5.48)	0.02

Δ- variable value changes during the observation period, expressed as a percentage of baseline values

The distribution of rs5498 genotypes in patients with T2DM and the control group are presented in Table 3. There are no statistically significant differences in the distribution of genotypes in patients with T2DM and the control group. The distribution of genotypes in the population of patients with T2DM was in Hardy-Weinberg equilibrium (SB2: χ2 = 0.83; *p* = 0.36; healthy controls: χ2 = 0.82; *p* = 0.36).

**Table 3:** Distribution of rs5498 genotypes for ICAM-1 in patients with T2DM and control subjects without T2DM

	Patients with T2DM *n* = 595	Subjects without T2DM *n* = 200	*p*
KK genotype	172 (28.9)	59 (29.5)	0.87
EK genotype	306 (51.4)	105 (52.5)	
EE genotype	117 (19.7)	36 (18.0)	
K allele	650 (54.6)	223 (55.8)	0.69
E allele	540 (45.4)	177 (44.2)	

Ultrasound markers of carotid artery atherosclerosis in patients with T2DM were compared between the first and the second examination in comparison to the rs5498 genotypes. There was a statistically significant difference in the annual increment of CIMT with regard to rs5498-ICAM genotypes, i.e. subjects with T2DM with the EE genotype had the biggest enlargment of CIMT per year in comparison with other genotypes (Table 4). We did not demonstrate, however, a statistically significant differences in subclinical markers of carotid atherosclerosis with regard to rs5498-ICAM genotypes in subjects without T2DM (Table 4).

**Table 4:** Changes in ultrasound markers of carotid artery atherosclerosis in subjects with T2DM and without T2DM between the first and second ultrasound examination of the carotid arteries according to rs5498 ICAM genotypes

	KK genotype	KE genotype	EE genotype	*p*
Subjects with T2DM				
Annual CIMT increment (μm/year)	12.76 (5.26–23.96)	20.34 (12.50–28.04)	26.09 (20.69–32.42)	0.04
Δ number of plaque segments	1.0 (0.5–3.0)	2.0 (1.0–2.5)	3.0 (2.0–3.0)	0.65
Δ sum of the plaques thickness (mm)	4.3 (1.4–7.8)	5.4 (1.55–10.15)	6.3 (2.3–8.1)	0.62
Subjects without T2DM				
Annual CIMT increment (μm/year)	10.22 (6.72–17.85)	13.47 (10.24–19.66)	16.46 (10.32–21.82)	0.12
Δ number of plaque segments	1.0 (0.5–3.0)	2.0 (1.0–3.0)	2.0 (1.0–3.0)	0.42
Δ sum of the plaques thickness (mm)	2.8 (1.3–6.2)	3.8 (1.40–6.08)	4.5 (1.8–6.64	0.58

Δ- variable value changes during the observation period, expressed as a percentage of baseline values

Table 5 demonstrates the effect of rs5498 genotypes on progression of markers of carotid atherosclerosis in patients with T2DM due to statin treatment. We demonstrated the EE genotype of the rs5498 to be associated with higher statistically significant annual increment of CIMT in subjects with T2DM that were not on statin treatment.

**Table 5:** Association of ICAM-1 polymorphism with carotid atherosclerosis progression in patients with T2DM due to statin treatment

	Statin treatment	KK genotype	KE genotype	EE genotype	*p*
Annual CIMT increment (μm/year)	+	11.77 (6.24–16.19)	18.06 (14.45–23.08)	22.62 (17.81–29.48)	0.13
	-	14.27 (10.22–19.32)	21.28 (15.21–28.27)	28.04 (24.32–32.56)	0.04
Δ sum of the plaques thickness (mm)	+	3.76 (2.8–5.40)	4.69 (3.68–5.12)	5.86 (4.16–7.68)	0.30
	-	4.92 (3.76–6.48)	6.40 (4.66–8.02)	6.93 (4.76–8.31)	0.74
Δ number of plaque segments	+	1.0 (0.5–2.0)	1.5 (1.0–3.0)	2.0 (1.0–3.0)	0.23
	-	1.0 (0.5–2.0)	2.0 (1.0–3.0)	3.0 (0.5–3.0)	0.57

Table 6 shows the relation in incidence of unstable plaques in patients with T2DM, adjusted to the classical risk factors for atherosclerosis and genotypes K469E polymorphism for ICAM 1 upon the inclusion in the study. When adjusted to other risk facotors, the rs5498 EE genotype had an independent effect on the presence of unstable plaques on the carotid artery.

**Table 6:** Adjusted analysis of rs5498-ICAM gene polymorphism and the presence of plaques/unstable plaques in the carotid arteries in patients with T2DM upon the inclusion in the study

	Presence of the plaques		Presence of the unstable plaques	
	OR (95 % CI)	*p*	OR (95 % CI)	*p*
Hypertension (0 = no; 1 = yes)	2.43	0.25	1.56	0.38
Systolic pressure (mmHg)	0.12	0.44	0.17	0.37
Serum LDL (mmol/L)	1.45	0.32	1.22	0.71
Serum HDL (mmol/L)	0.15	0.03	0.48	0.76
Hba1c (%)	0.72	0.10	1.14	0.49
KE	0.97	0.49	0.74	0.17
EE	0.85	0.51	1.69	0.03

All models were adjusted by age, sex, smoking, and treatment with statins. The reference group are homozygotes for allele K

Table 7 shows the results of multiple linear regression, adjusted to classic risk factors for atherosclerosis and rs5498-ICAM genotypes. When adjusted to other risk factors, the rs5498 EE genotype had an independent effect on the annual enlargement of CIMT in subjects with T2DM.

**Table 7:** Relation between genotypes K469E polymorphism for ICAM 1 gene and the progress of carotid artery atherosclerosis in patients with T2DM

	ΔCIMT/year		Δ Plaques segments		Δ Sum of the plaques thickness	
	β	*p*	β	*p*	β	*p*
Hypertension (0 = no; 1 = yes)	1.45	0.35	1.26	0.64	1.46	0.92
Systolic pressure (mmHg)	0.12	0.44	0.08	0.32	0.45	0.48
Serum LDL (mmol/L)	0.32	0.40	0.07	0.69	0.53	0.41
Serum HDL (mmol/L)	−0.04	0.18	−0.51	0.34	−0.39	0.90
Hba1c (%)	0.89	0.70	0.13	0.43	0.68	0.28
KE	1.37	0.49	0.74	0.17	1.14	0.37
EE	3.69	0.03	0.25	0.51	1.31	0.62

All models were adjusted by age, sex, smoking, and treatment with statins

The reference group are homozygotes for allele K

## Discussion

An association between the rs5498 polymorphism of the ICAM-1 gene and the progression of carotid atherosclerosis in subjects with T2DM was demonstrated in the present study. We found an association between the EE genotype of rs5498-ICAM-1 and the annual increment of CIMT. Moeover, a regression analysis demonstrated an association between the EE genotype of rs5498-ICAM-1 and the presence of unstable plaques in the carotid arteries. Our study is the first to establish an association between the rs5498-ICAM-1and the progression of carotid atherosclerosis. ICAM-1, produced by endothelial cells during inflammatory reactions, is involved in the adhesion reaction of monocytes, macrophages, T lymphocytes, and platelets to each other and to the vessel wall, which all favour the atherosclerotic process [21]. Moreover, ICAM-1 is involved in the interaction through intergrin receptors on leukocytes which enabled the binding to endothelial cells and transendotelial migration of leukocytes in the intima and leukocytes accumulations in vascular wall [14].

Several studies support the hypothesis that the ICAM 1 might influence the development of atherosclerosis. Pola and co-workers demonstrated that the EE genotype of the rs5498 was twice as frequent in patients with CVI than in the control group, indicating the the rs5498 was a genetic risk for ischemic stroke [22]. The association between the rs5498 polymorphism and coronary heart disease was not demonstrated in Caucasians in Ireland and Slovenia [19, 23]. In epidemiological studies in a seemingly healthy population, elevated levels of sl ICAM-1 were reported to be associated with an increased risk for the development of cardiovascular disease, periferal vascular disease, carotid artery disease and cerebrovascular stroke [7, 13, 24, 25]. Tang and co-workers reported that the level of serum ICAM-1 was associated with calcium score as a marker of coronary atherosclerosis. Moreover, they concluded that sICAM-1 might be a biomarker for coronary atherosclerosis [26]. Gaetan et al. in the Italian population and Shaker et al. in the Egyptian population reported that the EE genotype of the rs5498 was significantly more frequent in patients with peripheral arterial oclusive disease than in the control group [27, 28].

Finally, in the study we demonstrated the EE genotype of the rs5498 to be associated with higher statistically significant annual increment of CIMT in subjects with T2DM that were not on statin treatment.

The rs5498-ICAM-1 polymorphism was reported to be associated with an increased expression of ICAM-1 in our study in which subjects with T2DM with diabetic retinopathy were enrolled [29]. Namely, significantly higher sICAM-1 serum levels were demonstrated in Caucasian subjects with T2DM with the EE genotype compared with those with other (EK + KK) genotypes (918 ± 104 vs. 664 ± 209 microg/L; *P* = 0.001) [29]. We presume that the inceased expression of ICAM-1 might lead to an increased leukocytes adhesion to the vessel wall endothelium and the atherosclerotic process [30]. In their study, De Graba et al. found an increased expression of ICAM-1 in symptomatic in comparison to asymptomatic plaques [31]. Contrary to this report, however, Nuotio and co-workers did not report about an increased expression of ICAM 1, VACAM 1, E-selectin and P-selectin in symptomatic carotid plaques [32]. The rs5498 of the ICAM-1 gene results in a change in the amino acid sequence of the immunoglobulin-like domain 5. This domain is of crucial importance for the activity of the ICAM-1 protein [33]. We speculate that the EE genotype might affect the development of atherosclerotic markers via increased sICAM-1 serum activity.

## Conclusion

In our study, the EE genotype of the rs5498 of the ICAM-1 gene was associated with a more rapid progression of carotid atherosclerosis in subjects with T2DM in comparison with other genotypes.

## Statement of human and animal rights

All procedures followed were in accordance with the ethical standards of the responsible committee on human experimentation (institutional and national) and with the Helsinki Declaration of 1975, as revised in 2008.

## Statement of informed consent

Informed consent was obtained from all patients for being included in the study.
